# Radiographers’ perspectives on interactional processes during older persons diagnostic medical imaging encounters: a qualitative study

**DOI:** 10.1186/s12877-024-04792-x

**Published:** 2024-02-28

**Authors:** Kevin Ding, Chandra Makanjee

**Affiliations:** Department of Medical Radiation Science, University of Canberra, University Drive, 2617 Bruce, ACT Australia

**Keywords:** Older patients, Interactional processes, Medical imaging encounters, Qualitative study, Radiographers

## Abstract

**Background:**

Within a diagnostic medical imaging context, an interaction encompasses communication, physical contact and emotional support. These intricacies are an integral part in achieving a successful medical imaging outcome. An increasing ageing population presents unique challenges and leads to a higher demand for medical imaging services. There is a paucity of literature exploring the specialised knowledge and skills required by radiographers to service optimal person-centred care for elderly patients. The purpose of the study was to explore radiographers’ perspectives on interactional processes during older persons diagnostic medical imaging encounters.

**Methods:**

The study used a qualitative exploratory research design with a descriptive approach to gain insights from 12 purposively sampled Australian radiographers, through open-ended interviews conducted online or by telephone. Verbatim transcripts were produced, and a thematic analysis employed until data saturation had been reached.

**Results:**

The three themes that emerged from the data analysis were: (1) optimising care and communication, (2) expectations and preconceptions and (3) physical and emotional comfort and safety. Generally, the approach to undertaking older persons examinations entailed more adaptive and flexible competencies and skills in comparison to the familiarised routine diagnostic medical imaging encounters with the younger cohort. Radiographers shared aspects on striking a balance between efficiency and proficiency with the elderly patient needs, preferences, values, safety and well-being considerations. This required swift, complex decision-making and judgement calls due to the unpredictable nature of the context in which the elderly person was situated. The result was the adaptation of examination protocols through equipment manipulation, with minimal disruptions to emotional and physical comfort, achieved through interventions and support strategies.

**Conclusion:**

The results highlight the many considerations for radiographers during a short clinical interaction. There is optimism in adding value to the elderly persons experience through a complex interactional process. It is anticipated that the identified skills will inform on best practice principles to achieve an elderly person-centred care medical imaging outcome.

**Supplementary Information:**

The online version contains supplementary material available at 10.1186/s12877-024-04792-x.

## Background

Including Australia, most developed countries have an increasing ageing population, with the proportion of people aged 60 years and older to reach 2.1 billion globally by 2050 [1]. Rising life expectancies due to advancements in education, medicine and the economy has resulted in patients living longer, yet with chronic illnesses [2]. Evidence shows that an ageing population is more prone to chronic health conditions due to functional, physiological, psychological and social changes [3]. Those aged 75 and older seeking healthcare services typically present with unique challenges, such as physical limitations and reduced communication [4]. Such impairments may inhibit their ability to express their concerns and limit their responsiveness to environmental cues [4]. For these reasons, obtaining medical information from an ageing population can prove to be challenging [5].

Establishing rapport and maintaining effective relationships through communication are recognised as important components of the interactional processes between healthcare professionals and patients [6]. As part of the multidisciplinary team, radiographers are expected to deliver individualised quality person-centred medical imaging services. For the elderly person referred for a medical imaging examination, it is expected that services will be tailored to align with their choices, needs and preferences [7]. Given the brief and focused nature of general radiographic examinations, radiographers are required to effectively convey information, provide opportunities for information exchange and instruct the patient using both verbal and non-verbal cues, along with physical manipulation of the body part region of interest and equipment [6].

The number of medical imaging examinations for the general population was 0.94 per ED visit in 2014 [8]. While general radiographic examinations remain the cornerstone for the diagnosis of conditions experienced by the ageing population [9], little is known about the radiographers’ approach in delivering such services. To bridge this knowledge gap and improve on best practice principles, this study attempted to explore radiographers’ perspectives on interactional processes during geriatric diagnostic medical imaging encounters. It was envisaged that information regarding this population within medical imaging encounters could provide a better basis in designing quality services.

In the provision of medical imaging services, the approach to caring for frail and vulnerable elderly patients may differ from other age groups [7]. The diagnostic imaging of elderly patients requires specialised knowledge, as it can sometimes be difficult to distinguish between the natural ageing process and disease [10]. For example, a qualitative cross-sectional study established that inexperienced radiographers struggled to communicate or modify imaging techniques to suit patients with dementia [11]. Similarly, the risk of obtaining suboptimal pelvic radiographs increased for patients aged 60 years and older [12].

The aim of this study was to explore and gain insights into radiographers’ experiences, perspectives and understanding on interactional processes as a key determinant in optimising care throughout the elderly patient diagnostic medical imaging encounter. The assumption is that such experiences would identify elderly person-specific strategies and techniques, which, in turn, will inform on attributes and best practice principles that contribute towards value-added diagnostic medical imaging outcomes.

## Methods

This study is part of a larger research project focusing on the perspectives of the elderly population group and other healthcare professionals who are involved either directly or indirectly in offering diagnostic medical imaging services. The study design and findings have been reported according to the Consolidated Criteria for Reporting Qualitative Research (COREQ) checklist [13].

### Study design

For the purposes of the study, a qualitative exploratory research design was applied, using a descriptive approach [14]. The goal was to derive meaningful insights and understandings into the lived experiences of radiographers during their interactional processes with an elderly person referred for a diagnostic medical imaging examination. The primary characteristic of qualitative descriptive approaches is the straightforward representation of participant responses to explore certain events, interventions or phenomena [15]. The study was approved by the Human Research Ethics Committee, Faculty of Health, University of Canberra (HREC 2022–11,729).

### Participants and recruitment process

Australia’s health system is a complex mix of service providers and other healthcare professionals from a diverse range of organisations. The government and non-government sector collectively work to meet the national healthcare needs [16]. Within the Australian health system, diagnostic imaging has become increasingly popular, with policies focused on allowing patient access to such services [17]. Participants were recruited using non-probability purposive sampling techniques, as these allowed information-rich accounts of ageing population medical imaging encounters to emerge from a target audience [18]. To participate in this study, radiographers were qualified, based in Australia and involved in elderly population diagnostic medical imaging encounters. The exclusion criteria included students and other healthcare professionals. The recruitment plan involved the identification of prospective participants through professional networking opportunities during clinical placements. Participants of interest for this study were based in public hospital medical imaging settings and private clinics. Prospective participants were invited via emails with an attachment of the information leaflet and consent form. Once these participants agreed, an interview was scheduled at a time convenient to them.

### Data collection and analysis

Data collection occurred between July 2022 and February 2023 and involved individual, open-ended interviews conducted online or by telephone with radiographers practicing in private and public medical imaging settings in Australia. An interview guide was developed from literature [7, 10–12] and clinical experience. It consisted of open-ended questions with probes on participants’ experiences of elderly patient diagnostic medical imaging encounters, delivery of person-centred care, appropriate communication styles and suggestions for the emerging cohort of radiographers (see attached supplementary file 1). The intent behind the interview guide was to allow participant perspectives to emerge [19]. Piloting the interview was undertaken by three diagnostic radiographers: one from an academic background and the others from the private and public sector. They were interviewed for the purpose of providing feedback and incorporating their inputs through amendments made accordingly. These individuals were not a part of the study.

The first author (KD) conducted the online digital audio recorded interviews, which were verbatim transcribed and stored on a password-protected computer. These interviews lasted approximately between 20 and 30 min. Data analysis and interpretation of the transcribed data followed, which involved reading and re-reading the transcripts to immerse in the participants’ accounts and identifying repeated ideas or patterns to develop codes and categories. This process was guided by the staged approach described for using a thematic analysis framework [20]. The initial analysis and interpretation was conducted independently by both authors. The differences and similarities of the identified codes and categories were then discussed until an agreement was reached. Participants were then offered an opportunity to review, revise and validate the codes and categories. The participants agreed and there were no further changes. KD completed the thematic analysis of the data. This ongoing iterative analysis and interpretation of the transcripts informed the sample size, as interview continued until no recurring themes or subthemes emerged; this was the point of data saturation [21]. Throughout these described processes, no data analysis software was utilised, which allowed immersion into the data [22]. To further protect the anonymity of participants, codes were assigned to individual responses from P1-P12, where P is the participant and the numbers indicate the order in which they were interviewed.

### Quality of study

Despite being familiar with the medical imaging examination, KD spent at least a month at the research site to become further acquainted with the clinical environment. There was a specific focus on elderly patients referred for diagnostic medical imaging examinations to gain a better understanding and acquire rich, insightful data [23]. Peer debriefing and respondent validation were strategies to ensure credibility [24]. Direct quotations and thick descriptions to contextualise participant experiences confirmed transferability [24]. Documentation of data collection and analysis through an audit trail established dependability and confirmability [25].

### Reflexivity

Reflexivity refers to the self-awareness that researchers have on their own biases and influences during the collection and interpretation of data [26]. In this study, both investigators were medical radiation practitioners, with KD having a fair amount of clinical work experience and involvement in qualitative research. The second author (CM) is a diagnostic medical imaging person-centred care and qualitative research field expert, with extensive academic and clinical experience. Both authors did not have any affiliations with the clinical practice and or participants who took part in this study. The exception was to fulfill the purposes of the research through consenting, data collection and member checking. The capturing and collection of data was completed by KD. CM was responsible for quality assuring the research process and ensuring the authenticity of the captured data. As mentioned earlier, KD then completed the thematic analysis.

## Results

This study included 12 participants, with an equal number of males and females and ages ranging from 18 to over 60. Ten participants were based in public hospital medical imaging settings, while two were in private clinics. Their clinical experience varied between 4 and 44 years.

**Fig. 1:**
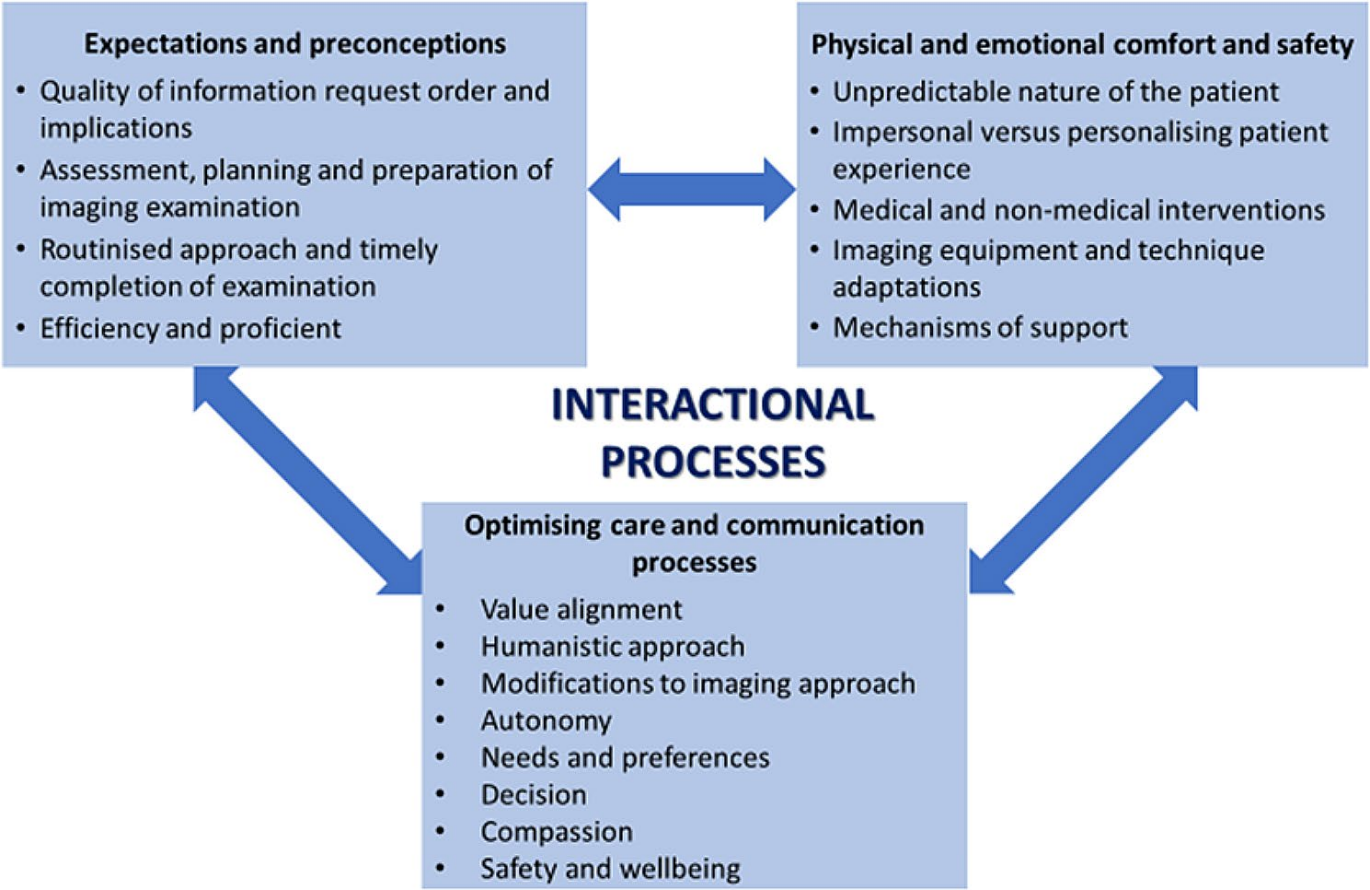
At the centre is the overarching theme representing the interactional processes with the subthemes, their respective categories and interrelated nature regarding an elderly medical imaging encounter

Following a thematic analysis framework process of the comprehensive data [20], the categories were overlapping and challenging to draw a clear distinction between them [27]. Attempts were made to primarily organise and identify overarching themes. The themes that emerged were: expectations and preconceptions, physical and emotional comfort and safety and optimising care and communication processes. These themes were interdependent and related due to the integrated nature of the diagnostic medical imaging encounter [28], as illustrated in Fig. 1.

### Expectations and preconceptions

This theme uncovered how the subjective nature of attitudes and assumptions can influence the delivery of personalised care and communication that are tailored to the unique circumstances of elderly patients. Based on the insights provided by participants, some radiographers demonstrated indifference towards elderly patients owing to the frequency of their medical imaging encounters, while others displayed enthusiasm and had positive expectations.

*“Just because they’re older, it doesn’t mean you should treat them any less, which I know happens sometimes, because it’s like, ‘Oh, another neck of femur fracture,’ and then just doing it.”* (P4).

*“My overall experience would be quite positive. I find older people probably easier to talk to, just asking them general questions… they really appreciate that and I appreciate their answers back. I just find them easier to communicate with and I enjoy listening what they have to say to me.”* (P11).

An initial assessment of mobility is essential for determining the level of involvement required from radiographers during geriatric diagnostic medical imaging encounters. Participants were able to predict the trajectory of the examination by considering geriatric patient presentation.

*“In my experience, I assess the patient and I know how… to do the pictures that are good for me but are also going to be okay for the patient. Some pictures you know straight away. For example, I won’t be able to ask this patient to lie on their side… So, in your mind, you are already thinking, ‘Okay, I have to do a horizontal beam X-ray for this knee.’ So that’s the way you have to think… So, I have experienced as well– I take the first picture… and if I see something that is like, you know, fracture of the hip, sometimes instead of lying the patient on their side, I do my horizontal beam view.”* (P2).

Effective care and communication provided to elderly patients can also be impacted by the quality of radiology request forms from requesting physicians. One participant deemed adequate clinical information critical in the making of clinical judgements.

*“If you get, basically what I say garbage in, garbage out, if you get a rubbish request, then you’re going to get a bit of a… not great X-ray and rubbish report as well. So yeah, we need to know why it is that we’re doing it. We need to know more information about maybe the history of the patient, how long they have had that pain for, that kind of stuff, because that will then also tell you how you’re going to be able to position them in the first place. Like, are you going to need a certain way and then obviously, what you’re going to do with that information once you’ve got the X-ray and the report’s been done. Is it going to change our management? Is it answering the clinical question? So that’s yeah, very important.”* (P3).

### Physical and emotional comfort and safety

The next theme shows that creating a comfortable and safe environment for elderly patients is an intended consequence of the provision of high-quality care and communication despite external influences. One participant underscored the obligation for radiographers to consider an elderly patient’s level of pain, suggesting a delay of examination to minimise further discomfort.

*“With an elderly patient… [who] clinically appears to have a neck of femur fracture and is in an irregular amount of pain, it might be worth it to delay the examination. I had a patient recently who did exactly that… I sent the patient back to the referring doctor and found out that they were due for a nerve block. They didn’t do it yet, so a classic example of a patient who could have had their second, further views done with relative comfort.”* (P9).

Many participants highlighted how small gestures of compassion, such as providing blankets, pillows and protecting dignity can play a crucial role in managing the discomfort of elderly patients.

*“So, what I did was I actually… got some towels and she felt a bit faint, so I had… a blanket for her… and I actually just sat down next to her… I put a blanket over her and just a pillow and I just let her have a lie down there… and was just talking about general things like her grandkids or the weather and I stayed with her for five to ten minutes because I had the time.”* (P6).

Safety in geriatric diagnostic medical imaging encounters was a multidimensional and multifaceted construct that varied in terms of its components and manifestations. Some participants used their perceptiveness to mitigate fall-related risks whereas others developed a more holistic approach in considering their own safety as radiographers.

*“… the other day, we had a patient who wasn’t too good on her feet. We tried to stand-transfer across to the CT table… I noticed that she was wearing socks instead of just her bare feet, so we popped her back into bed because obviously, that’s a risk… because she can slip in her socks.”* (P5).

*“… when it comes to patients that become aggressive or refuses to do stuff… like, punch us or get really aggressive… if it’s like putting our safety at risk, we definitely shouldn’t push on. Something we should look to do is contact the referrer… see if they want to continue… or perhaps… give them some sedation first and then get them back down a bit later.”* (P10).

### Optimising care and communication

This theme explains how participants noticed their strong preference for human connection during medical imaging encounters. This included a desire to share personal stories to connect with radiographers on a more personal level. Most participants reported these interactions to be mutually beneficial, as they helped to establish a sense of rapport and trust.

*“… I find that the ageing population values different aspects of care than a younger generation. The younger generation will want a fast, quick service with quick results whereas an ageing population care more about the human interaction.”* (P6).

One participant warned that while elderly patients appreciate personal interactions, time constraints in a busy medical imaging department can make it challenging to accommodate such conversations.

*“… elderly patients are often lonely and sometimes they just want to chat and… it presents a challenge to the radiographer because in a busy ED environment, you don’t always have time for a chat… that balance is a difficult challenge in itself.”* (P9).

Given the hearing difficulties, neurodegeneration and physical immobility that often accompany elderly patients, several participants highlighted the necessity for modifications to standard practices in order to optimise care.

*“… sometimes even when they’ve got hearing difficulties… demonstrating what position I want them. So, while they’re sitting in the bed and I want them to stand up, I’ll explain, ‘Okay, I need you standing up like this and put your chest on the board like this…’”* (P8).

Participants spoke of the impact of dementia on interactional processes with elderly patients. One conveyed the potentially confusing and disorienting journey through a medical imaging encounter for elderly patients with dementia.

*“… if they fell at home and they’ve got dementia, they are in pain but don’t know why and they’re suddenly in a building that they’ve never been to before and it’s all very bright, lots of people are coming in, poking them, so it’s about speaking to them respectfully while also acknowledging that they might be going through a traumatic experience.”* (P3).

However, participants cautioned against overgeneralising and assuming that adaptive techniques are required for all geriatric diagnostic medical imaging encounters. For instance, infantilizing language is situational and may be perceived as condescending in some cases.

*“But it’s a fine line because you don’t really want to do any baby talk to older people and make them feel dumb because that can be quite offensive to them.”* (P8).

Building on the previous subtheme of individualised needs and preferences, participants also emphasised the importance of compassion and empathy in promoting elderly patient satisfaction. Many participants described a relational approach in which ageing patients were imagined to be a close family member.

*“Every time I do an old patient… I think of my mother… So, I think I would like someone to see my mother, you know, to take care of her the same way as taking care of the elderly.”* (P2).

Participants stressed the significance of employing verbal and written communication strategies that empower elderly patients to actively engage in their own decision-making processes and promote autonomy.

*“For people that can’t hear and they’re in their old age, sometimes I will write down what I want them to do. I will have a few cards for them to point to, ‘Yes,’ ‘No,’ ‘I’m not sure.’”* (P6).

*“Always giving them the ability to say yes or no. Some people hardly wait for the patient to say yes before they’re like, setting up for the X-ray… make sure they fully understand before you continue.”* (P8).

A significant point of reference in providing emotional support is through the active involvement of carers and nurses during geriatric diagnostic medical imaging encounters. One participant also expressed an anticipation for additional resources in order to support the needs of an elderly patient.

*“I might wait for another radiographer to get back from wherever they are before I call the patient around because I don’t want to be in a position putting either myself or the patient at risk, as they don’t really have the resources there, like getting them on or off the table.”* (P9).

*… I would say having their nurses come down or like a family member accompanying them really helps because they would have a familiar face that they associate with and more likely to be compliant with for the examination.”* (P10).

Overall, the experiences shared by participants revealed the complex nature of interactional processes during geriatric diagnostic medical imaging encounters. There are various external factors that can impact the quality of care and communication, some of which may be unforeseeable or difficult to predict.

## Discussion

This study aimed to explore the viewpoints of radiographers on interactional processes during elderly diagnostic medical imaging encounters. It was anticipated that such insights could contribute to improved outcomes in diagnostic medical imaging for the ageing population. The findings suggested strategies to optimise care and communication, influencing expectations and preconceptions and physical and emotional comfort and safety outcomes.

The consensus among participants was that elderly patients have a natural inclination to seek personalised human interaction during their medical imaging encounters. This finding is consistent with previous research indicating the effectiveness of personalised interventions to improve the health outcomes of the ageing population [29]. Compassionate care can be achieved through targeted actions and communication strategies to address one’s vulnerability [30]. While participants recognised the importance of interactive processes, tensions arose when balancing efficiency and the quality of care. In a busy medical imaging department limited by time constraints, trivial remarks as part of radiographers’ communication have been found to be situational and are likely to be ignored [1]. Moreover, participants identified several modifications to standard practices to overcome challenges associated with the neurodegeneration and physical immobility of some elderly patients. Several studies have supported the reliance on adaptive techniques to assist with the positioning of patients, while maintaining comfort and imaging quality [31–32]. Building upon the idea of modifications to standard practices, some participants pointed out that it is equally important not to assume all elderly patients have the same needs and preferences. One study warned against infantilising language, as the ageing population may perceive childlike words and expressions to be demeaning [33]. Instead, a person-centred approach is one that empowers patients by facilitating an ideal experience to achieve their goals [34]. With the acknowledgement that individualised care cannot be treated in isolation from overall medical imaging processes and procedures, participants highlighted the necessity to support the autonomy and decision-making of elderly patients. Research has shown that patients are eager to actively participate in their healthcare journey and shared decision-making can lead to improved outcomes and satisfaction [35–36]. An additional consideration for participants was the caring of elderly patients through multidisciplinary teamwork. In this regard, carers and nurses were deemed to be essential members of the team, given their familiarity with their respective patient. Effective teamwork between healthcare professionals provides the opportunity for person-centred care and optimal patient outcomes [37].

Participants shared the impacts that pre-existing assumptions and beliefs have on their interactions with elderly patients. A study using focus group interviews as part of a qualitative methodology found that negative physical stereotypes associated with ageing have resulted in discriminatory biases among some healthcare professionals [38]. Such ageist beliefs can have significant implications on the mental and physical well-being of elderly patients, such as reduced confidence and less engagement in healthy behaviours [39]. In other words, radiographers have to recognise and challenge their expectations and preconceptions of elderly patients in order to provide an equitable and unbiased service. Participants emphasised that an initial assessment of patient mobility allowed for a clear direction in terms of positioning. Similarly, an observation of patient’s body habitus and region of interest may necessitate adjustments to obtain more projections [40]. Influencing external factors featured prominently in the responses of participants. One such factor was the quality of radiology request forms, which shaped the trajectory of elderly diagnostic medical imaging encounters. Conflicting or inadequate presentation of clinical information is recognised and widespread [41].

Participants expressed an emphasis on the importance of ensuring the physical and emotional comfort and safety of elderly patients. In this age group, common complaints of discomfort involved joint stiffness, pain and sensations of coldness [33]. Participants found that the offering of blankets and pillows was a solution to combat these issues. Elderly patients are also prone to fall-related injuries, which can pose unique difficulties for radiographers [33]. Participants minimised these risks through a visionary approach and by taking preventative measures.

**Fig. 2:**
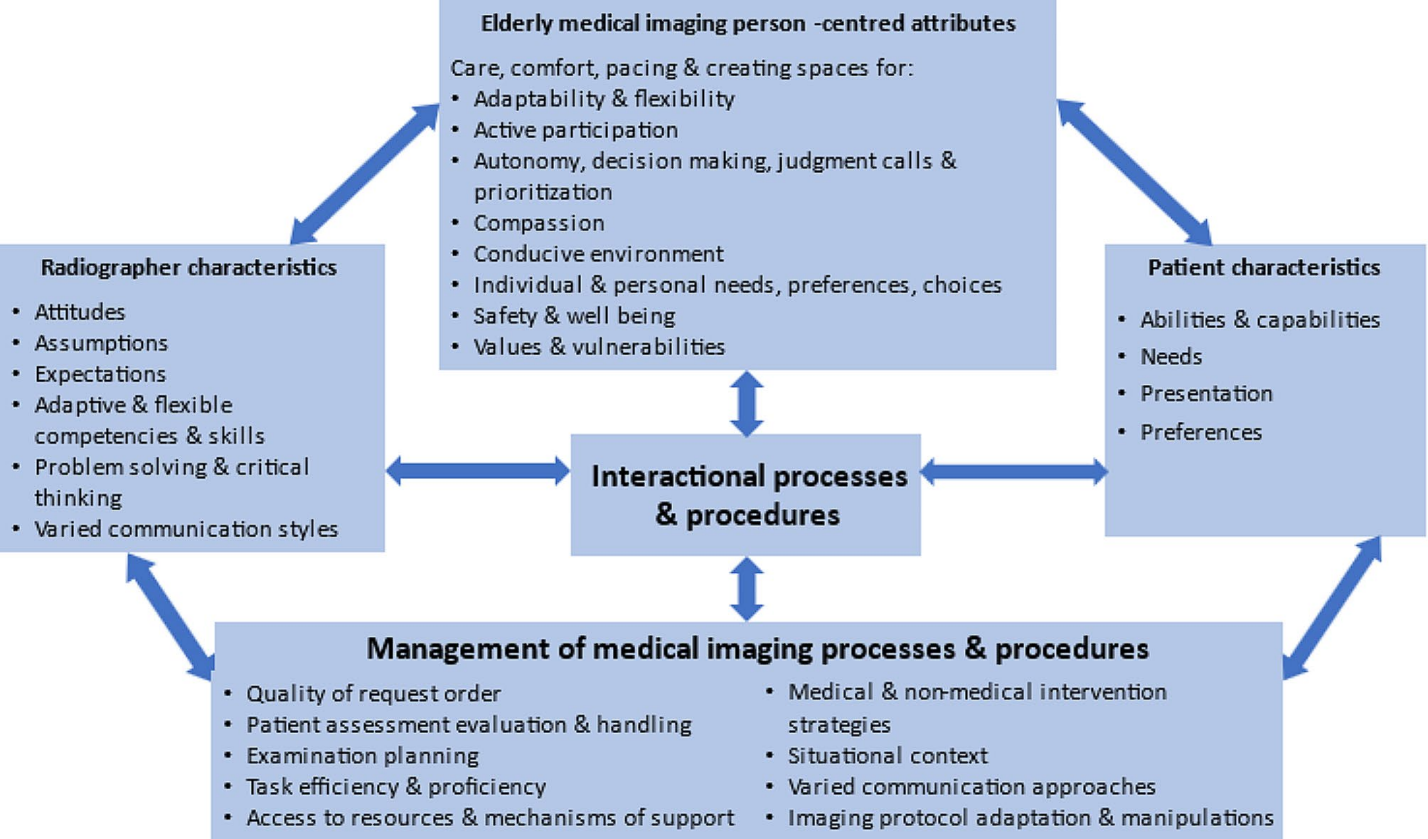
Proposed framework illustrating the interconnected nature and intricacies to consider in achieving a value-added interactional elderly person-centred diagnostic medical imaging outcome

In summary, the proposed framework captures the complex, interrelated and connected nature of the various aspects to consider in achieving high-quality, interaction-focused elderly person-centred diagnostic medical imaging outcomes (see Fig. 2).

With reference to Fig. 2, radiographer and patient characteristics and management processes enable a value-added, elderly person-centred, friendly diagnostic medical imaging examination. Maintaining diagnostic medical imaging safety measures with minimal disruptions to the well-being of elderly patients requires critical thinking, problem solving and judgement calls. Figure 2 aligns with achieving value-added diagnostic medical imaging outcomes. The complexities and intricacies of interactional processes highlighted by participants creates an awareness, encourages and empowers radiographers to enhance elderly-friendly diagnostic medical imaging encounters.

### Strengths and limitations

The primary strength of this study is the unique scientific contributions, such as the proposed framework which could serve as a guide for the considerations of the complexities involved in an elderly-focused diagnostic medical imaging encounter. Additionally, it illustrates the links between various elements of elderly patient management to quality assure a person-centred diagnostic medical imaging outcome. This was achieved through insights from clinical practice radiographers on their experiences and perspectives of their interactional processes with elderly patients. From a health system context, the incorporation of radiographers from both private and public clinical settings allowed for a comprehensive capturing of diverse perspectives on interactional processes. As a baseline, radiographers can take inspiration from the identified elderly person-centred strategies and adopt these into their own practice. The results will benefit radiographers and students to become more proficient in facilitating positive interactional processes and constructively switch between the quick-paced versus the slower-paced encounter to encourage active engagement. This requires quick, enhanced critical thinking and problem-solving capabilities and skills to manage elderly-focused diagnostic medical imaging encounters.

However, findings from qualitative research are context-specific and cannot be generalised beyond the sampled population. While the intention to use open-ended questions and probes in the interview guide allowed data to emerge from the targeted population group, some participants provided generic or superficial responses. Therefore, the collected data may not have fully captured the in-depth accounts of their experiences of diagnostic medical imaging encounters. Participants unable to meaningfully articulate could be due to a lack of research-related interview experiences [42], routinised undertaking of imaging examinations [43] or fatigue.

### Recommendations

Further review into the experiences and perspectives of elderly patients may be of benefit to optimise interactional processes during diagnostic medical imaging encounters. Focus group interviews could be utilised to gain comprehensive insights and compare the experiences of participants using a collaborative and interactive environment. To expand the scope of this study would be to research into the financial implications and cultural differences of older persons medical imaging encounters. Future research could focus on including other diverse healthcare professionals to become familiar with their guidelines and integrate imaging-specific strategies, special care and communication needs for elderly patients.

From a clinical practice context, radiographers are recommended to be inclusive and sensitive of the diverse needs of elderly patients to ensure a safe, high quality diagnostic medical imaging outcome. Professional development strategies should be established to equip radiographers with specific skill sets to manage elderly patients with cognitive and physical impairments. It will be useful to pick up on cues when elderly patients share their life experiences and expertise, identify any barriers and how they respond. For instance, devising interventions such as communication and or written aids.

From an educational perspective, diagnostic medical imaging courses could include a dedicated subject that integrates theoretical and practical components into clinical placements at a residential aged care facility. This would allow students to develop the required competencies and skills to obtain an understanding of the particular needs to foster effective engagement during diagnostic medical imaging encounters. A post graduate course in this specific area should also be considered. For radiographers, opportunities to attend courses on older person care and management should be readily accessible.

From a profession point of view, a diverse team of stakeholders should be established to collectively explore and review current practice-specific standards for the needs of various age categories. As a result, interventions that are currently in place can be identified and refined, or new ones created using an evidence-based approach. Robust discussions to employ multiskilled diagnostic medical imaging practitioners or expanding the scope of practice of assistant radiographers should take place.

## Conclusions

The quality of care received by elderly patients is heavily influenced by the interactional processes that occur in a medical imaging encounter. It is important to direct special attention towards optimising care and communication, challenging expectations and preconceptions and achieving emotional and physical comfort and safety for elderly patients. Rather than rely on the familiar quick-paced imaging, there must be an emphasis on proficiency and going above and beyond to deal with unexpected and unpredictable situations. The commitment to exceeding expectations thereby adds value to the elderly persons journey through the short, but complex diagnostic medical imaging encounter.

### Electronic supplementary material

Below is the link to the electronic supplementary material.

Supplementary Material 1

Supplementary Material 2

## Data Availability

The datasets generated and/or analysed during the current study are not publicly available due to the nature of a qualitative study but are available from the corresponding author on reasonable request.
