# Supplementary material for: Radiographers’ perspectives on interactional processes during older persons diagnostic medical imaging encounters: a qualitative study
Source: BMC Geriatr. 2024 Feb 28;24:205. doi: 10.1186/s12877-024-04792-x (PMC10900639; doi:10.1186/s12877-024-04792-x)
Supplement: Supplementary file 1 — Supplementary Material 1 [file 12877_2024_4792_MOESM1_ESM.docx]

**Interview schedule**

**Interview number:**

**Interviewee:**

**Interviewer:** Kevin Ding

**Date:**

**Meeting place:**

**Duration of interview:**

**Contact Information**

**Phone number:**

**Email:**

**Name of course/qualification/s:**

**Institution of qualification:**

**Institution: public or private**

**State:**

**Experience:** ______ years ______ months

**Modalities/specialisation:**

**Years of experience:**

**Gender:**

**Age category:**

**Reminders:**

- Check if the audio-recording device is working
- Thank participants for agreeing to participate
- Explain information leaflet
- Get consent
- SWITCH RECORDING EQUIPMENT “ON”

**Icebreaker:**

1. Tell me how your day is going.

**Possible questions if the issues are not flowing directly from the icebreaker:**

2. Could you share your experiences on ageing patients referred for diagnostic imaging investigations?

**If necessary, probe for:**

- How the quality of a referral impacts on the planning of imaging examinations and technique adaptions
- Patient care and communication aspects when undertaking medical imaging examinations
- Workplace culture, environment and physical characteristics

**Reminders:**

- Thank participants
- Switch off audio-recorder
- Note length of interview: ………………………... minutes

**Field notes and reflective notes:**
