# Supplementary material for: Radiographers’ perspectives on interactional processes during older persons diagnostic medical imaging encounters: a qualitative study
Source: BMC Geriatr. 2024 Feb 28;24:205. doi: 10.1186/s12877-024-04792-x (PMC10900639; doi:10.1186/s12877-024-04792-x)
Supplement: Supplementary file 2 — Supplementary Material 2 [file 12877_2024_4792_MOESM2_ESM.docx]

COREQ (COnsolidated criteria for REporting Qualitative research) Checklist

A checklist of items that should be included in reports of qualitative research. You must report the page number in your manuscript where you consider each of the items listed in this checklist. If you have not included this information, either revise your manuscript accordingly before submitting or note N/A.

| **Item No.** | **Topic** | **Guide Questions/Description** | **Reported on Page No.** |
| --- | --- | --- | --- |
| **Domain 1: RESEARCH TEAM AND REFLEXIVITY** | | | |
| Personal characteristics | | | |
| 1 | Interviewer/facilitator | KD conducted interviews | 7-8 |
| 2 | Credentials | KD- BMRS(Hons)  CM Brad (Hons), MRad, PhD Rad | NA |
| 3 | Occupation | KD -Clinical Medical Imagin Practitioner  CM – Snr Lecturer / Medical Imaging Practitioner | NA |
| 4 | Gender | KD – Male  CM- Female | NA |
| 5 | Experience and training | KD – 4 years clinical training and practitioner in medical imaging with relevant training and knowledge in qualitative research. Completed four units: Research in Health, research in medical radiation science, project design and report writing  CM – 37years experience in clinical practice in medical imaging with teaching and training and conducting research  Field expert in person centred medical imaging and research philosophy, design and methods. Reviewer for several journals with supervision experience of post graduate students and +25 publications. | NA |
| Relationship with participants G | | | |
| 6 | Relationship established | No | 8 |
| 7 | Participant knowledge of the interviewer | No | 8 |
| 8 | Interviewer characteristics | KD completed a unit in research methods, project design and report writing with research experience in conducting interviews and analysing qualitative data. | 8 |

|  |  |  |  |
| --- | --- | --- | --- |
| **DOMAIN 2: STUDY DESIGN** | | | |
| Theoretical framework | | | |
| 9 | Methodological orientation and Theory | A qualitive exploratory research design was used. A thematic approach to data analysis was used for the development of the manuscript. | 5 |
| Participant selection | | |  |
| 10 | Sampling | Purposive | 5 |
| 11 | Method of approach | Professional Network | 6 |
| 12 | Sample size | 12 |  |
| 13 | Non-participation | None of our participants declined participation. |  |
| Setting | | | |
| 14 | Setting of data collection | Interviews were conducted telephonically | 6 |
| 15 | Presence of non-participants | The researcher ensured that there were no bystanders by informing on the privacy and confidential nature of the interviews. |  |
| 16 | Description of sample | Non -Probability – purposive sampling was used to acquire rich insights. Exclusion criteria was stated | 5 |
| Data collection | | | |
| 17 | Interview guide | See supplementary file |  |
| 18 | Repeat interviews | No | NA |

| 19 | Audio/visual recording | All interviews were digitally recorded | |  |
| --- | --- | --- | --- | --- |
| 20 | Field notes | Yes | |  |
| 21 | Duration | 20-30minutes | |  |
| 22 | Data/ thematic saturation | Yes | |  |
| 23 | Transcripts returned | Yes | | 8 |
| **DOMAIN 3: ANALYSIS AND FINDINGS** | | | | |
| Data analysis | | | | |
| 24 | Number of data coders | 2 |  | |
| 25 | Description of the coding tree | Yes |  | |
| 26 | Derivation of themes | Yes |  | |
| 27 | Software | None |  | |
| 28 | Participant checking | Yes |  | |
| Reporting | | | | |
| 29 | Quotations presented | Yes | 10-16 | |
| 30 | Data and findings consistent | Yes |  | |
| 31 | Clarity of themes | Yes |  | |
| 32 | Clarity of minor themes | Yes | 10-16 | |
